# Supporting Carers of Children and Adolescents with Eating Disorders in Austria (SUCCEAT): Study protocol for a randomised controlled trial

**DOI:** 10.1002/erv.2600

**Published:** 2018-05-06

**Authors:** Claudia Franta, Julia Philipp, Karin Waldherr, Stefanie Truttmann, Elisabeth Merl, Gabriele Schöfbeck, Doris Koubek, Clarissa Laczkovics, Hartmut Imgart, Annika Zanko, Michael Zeiler, Janet Treasure, Andreas Karwautz, Gudrun Wagner

**Affiliations:** ^1^ Eating Disorders Unit, Department of Child and Adolescent Psychiatry Medical University of Vienna Vienna Austria; ^2^ Ferdinand Porsche Distance Learning University for Applied Sciences Vienna Austria; ^3^ Parkland‐Clinic Clinic for Psychosomatic Medicine and Psychotherapy Bad Wildungen‐Reinhardshausen Germany; ^4^ Department of Psychological Medicine, Section of Eating Disorders King's College London, Institute of Psychiatry, Psychology & Neuroscience London UK

**Keywords:** anorexia nervosa, carers, children and adolescents, skills training, motivational interviewing

## Abstract

Supporting Carers of Children and Adolescents with Eating Disorders in Austria (SUCCEAT) is an intervention for carers of children and adolescents with anorexia nervosa and atypical anorexia nervosa. This paper describes the study protocol for a randomised controlled trial including the process and economic evaluation. Carers are randomly allocated to one of the 2 SUCCEAT intervention formats, either 8 weekly 2‐hr workshop sessions (*n* = 48) or web‐based modules (*n* = 48), and compared with a nonrandomised control group (*n* = 48). SUCCEAT includes the cognitive‐interpersonal model, cognitive behavioural elements, and motivational interviewing. The goal is to provide support for carers to improve their own well‐being and to support their children. Outcome measures include carers' distress, anxiety, depression, expressed emotions, needs, motivation to change, experiences of caregiving, and skills. Further outcome measures are the patients' eating disorder symptoms, emotional problems, behavioural problems, quality of life, motivation to change, and perceived expressed emotions. These are measured before and after the intervention, and 1‐year follow‐up.

## 1 INTRODUCTION

Anorexia nervosa (AN) is a severe mental disorder with high mortality rates compared with other mental disorders (standardised mortality ratio = 5.9, 95% CI [4.2, 8.3]; Chesney, Goodwin, & Fazel, 2014). It is state of the art to involve carers (mostly parents) in the treatment of children and adolescents with eating disorders (EDs; National Institute for Clinical Excellence [NICE], 2017). Caring for a child who suffers from AN can have a serious emotional impact on the parents and lead to distress, anxiety, and depression (Kyriacou, Treasure, & Schmidt, 2008b; Whitney, Haigh, Weinman, & Treasure, 2007; Zabala, Macdonald, & Treasure, 2009).

On the one hand, previous studies have revealed that some parents are likely to show unhelpful behaviours for the patients' outcome, such as accommodating behaviours and dysfunctional interpersonal interactions. It is mostly the mothers of adolescent patients who spend more time involved in care compared with fathers, mainly providing food and emotional support; and they usually report higher levels of distress and accommodating behaviour (Rhind et al., 2016). Family members tend to show accommodating behaviours and reorganise their behaviours around the ED symptoms: For example, they accept meal rituals and low‐calorie foods and turn a blind eye to unwanted behaviours (Sepulveda, Kyriacou, & Treasure, 2009). When both parents are highly accommodating, these accommodating behaviours may maintain the illness and negatively influence the outcome of adolescents (Salerno, Rhind, Hibbs, Micali, Schmidt, Gowers, Macdonald, Goddard, Todd, Lo Coco, et al., 2016a). When the families' level of stress increases, family members are at risk of developing high expressed emotions (HEE; i.e., criticism and emotional overinvolvement; Sepulveda et al., 2010). Anxious parents, for example, might show emotional overinvolvement, which potentially prevents the patients' independence and inhibits their sense of self‐efficacy (Kyriacou, Treasure, & Schmidt, 2008a; Whitney et al., 2005).

On the other hand, there are families who seek help with their caregiving role (Haigh & Treasure, 2003; Zitarosa, de Zwaan, Pfeffer, & Graap, 2012). The importance of supportive interventions for the caregivers of someone with an ED has been demonstrated in various studies (Magill et al., 2016; Schwarte et al., 2017; Spettigue et al., 2015; Treasure et al., 2001). Family therapy has been proven to be effective with adolescent ED patients (Ciao, Accurso, Fitzsimmons‐Craft, Lock, & Le Grange, 2015; Eisler et al., 2016). Strategies to meet the needs of parents of patients with EDs aim to equip parents with knowledge and behaviour change skills. There are different approaches described in the literature: workshops for families (Whitney, Murphy, et al., 2012; Treasure, Whitaker, Todd, & Whitney, 2012), self‐care interventions with the guidance of experienced and trained carers including DVDs (Experienced Carers Helping Others, ECHO; Goddard, Macdonald, Sepulveda, et al., 2011; Goddard, Hibbs, et al., 2013; Goddard, Raenker, et al., 2013; Hibbs, Magill, et al., 2015; Magill et al., 2016), and intervention via the Internet (Grover, Naumann, et al., 2011; Grover, Williams, et al., 2011; Hoyle, Slater, Williams, Schmidt, & Wade, 2013). All of these approaches rely on “The New Maudsley Method” (Treasure, Schmidt, & Macdonald, 2010; Treasure, Smith, & Crane, 2007). The “New Maudsley Method” was developed for the adult carers of adult patients, and little work in this area has been carried out with the carers of adolescent patients. The ‘”New Maudsley Method” was found to reduce carers' HEE (emotional overinvolvement and criticism), distress, burden (Hibbs, Rhind, Leppanen, & Treasure, 2015), anxiety, negative experiences of caregiving (Grover, Naumann, et al., 2011), and time spent caregiving (Hibbs, Magill, et al., 2015). It was shown that the parents' improvement had positive effects on the patients with ED themselves. These included reduction of the ED psychopathology of patients with AN, a reduction of their inpatient bed days (Hibbs, Magill, et al., 2015), an improvement in their quality of life, and an increase in their weight gain (Goddard, Hibbs, et al., 2013; Whitney, Murphy, et al., 2012). Adult patients showed a positive attitude to involving parents in their care (Goddard, Macdonald, & Treasure, 2011).

The previous studies mostly included adult patients and fewer or no adolescent patients. Only one randomised controlled trial (RCT) has examined the effects of the self‐help intervention ECHO for parents of adolescent patients exclusively (13–21 years) so far (Rhind et al., 2014). Parents of adolescents received the training materials (a book and a set of DVDs), and coaches (individuals with experience in caregiving for someone with an ED) provided guidance via telephone. The parents of adolescents rated the intervention favourably and reported positive experiences on account of the ECHO intervention (Goodier et al., 2014). Optimising caregiving skills and reducing accommodating behaviours by the carers could aid the recovery of the patients (Salerno, Rhind, Hibbs, Micali, Schmidt, Gowers, Macdonald, Goddard, Todd, Lo Coco, et al., 2016a; Salerno, Rhind, Hibbs, Micali, Schmidt, Gowers, Macdonald, Goddard, Todd, Tchanturia, et al., 2016b).

A recent meta‐analysis of interventions for parents of children with EDs highlights a clear need for RCTs that include outcomes for the children and adolescents (Hibbs, Rhind, Leppanen, et al., 2015). In addition, there is also a need to make interventions for parents cost‐effective (Roick et al., 2001), available, and affordable (Fairburn & Patel, 2014).

Supporting Carers of Children and Adolescents with Eating Disorders in Austria (SUCCEAT) is based on “The New Maudsley Method” (MacDonald, Todd, Whitaker, Creasy, Langley, & Treasure, in prep.; Schmidt & Treasure, 2006; Treasure et al., 2010; Treasure, Smith, et al., 2007). For the SUCCEAT intervention, the material from “The New Maudsley Method” was adapted as a therapeutic 8‐week intervention for use with carers of children and adolescents (see Table 1). These were translated into German and also adapted to a German‐speaking cultural context (Treasure et al., in prep.). This is the first protocol for an RCT, which compares workshop support groups with web‐based support groups directly and also includes outcome of patients younger than 13 years (10–19 years).

**Table 1:** SUCCEAT intervention components based on the cognitive‐interpersonal maintenance model^a^

Content of eight SUCCEAT workshops sessions and web‐based modules		Aims
Interpersonal Factors	Aetiology of anorexia nervosa; explanation of relationship patterns: high expressed emotions, caring styles, and animal models	Knowledge about the illness, caring with just enough control, compassion, and consistency
Brain and Compassion	Effects of anorexia nervosa on the brain, thinking styles, obsessive‐compulsive personality traits, and self‐compassion	Recognising one's own thinking styles and personality traits, increasing flexibility, and being self‐compassionate
Stages of Change	Transtheoretical Model of change (Prochaska & DiClemente, 1984), eliciting confidence, importance, and readiness to change	Recognizing the different stages of change and supporting changes in these different stages
Motivational Interviewing I	Introduction to Motivational Interviewing (Miller & Rollnick, 2002), main principles of reflective listening and communication	Expressing empathy, motivating change, rolling with resistance, and supporting self‐efficacy
Motivational Interviewing II	Enhancement of Motivational Interviewing, communication tools: reflection, reframing, etc.	Initiating change talk (refers to desire, ability, reasons, and needs to change) and avoiding communication traps
Stress and Emotions	Improving emotional intelligence and managing personality traits (perfectionism and avoidance)	Supporting emotional self‐regulation
Difficult Behaviours	Coping with difficult behaviours, elements of cognitive behavioural therapy: Antecedent–Behaviour–Consequence (ABC model; Ellis, 1973)	Changing antecedents, behaviours, and consequences
Stage of Maintenance	Recognising recovery, complete remission, and partial remission; crisis management	Preventing relapses
Additional material offered to the carers		
DVD^b^	Instructions explain communication skills and animal models Role play scenarios show examples for disadvantageous and advantageous communication Audio files give examples of providing meal support	
SUCCEAT workbook	Summarises and extends the contents of the intervention, and contains further information and exercises One chapter informs the patients about the intervention	

*Note*. SUCCEAT = Supporting Carers of Children and Adolescents with Eating Disorders in Austria.

a MacDonald, Todd, Whitaker, Creasy, Langley & Treasure, in prep.; Treasure et al. (2010); Treasure, Smith, et al. (2007); Schmidt and Treasure (2006); and Treasure and Schmidt (2013).

b *How to Care for Someone with an Eating Disorder* (The Succeed Foundation, 2011; German translation of our research group).

### 1.1 Theoretical background of the SUCCEAT intervention

The SUCCEAT intervention highlights several benefits of “The New Maudsley Method” (Treasure, Smith, et al., 2007; Treasure et al., 2010; Treasure & Nazar, 2016). “The New Maudsley Method” consists of psychoeducation (e.g., aetiology of EDs, the effects of starvation on the brain, emotional self‐regulation, and personality traits) and communication tools; and it incorporates different theoretical models:

- Motivational Interviewing (MI; Miller & Rollnick, 2002),
- the cognitive‐interpersonal maintenance model (Goddard, Salerno, et al., 2013; Schmidt & Treasure, 2006; Treasure & Schmidt, 2013),
- the Antecedent–Behaviour–Consequence model (ABC model; Ellis, 1973), and
- the Transtheoretical Model of Change (Prochaska & DiClemente, 1984).

Besides the focus on MI in two workshop sessions and web‐based modules, respectively (see Table 1), the whole SUCCEAT intervention provides skills for behaviour change through an MI framework (Miller & Rollnick, 2002; Treasure et al., 2012). The coaches from the SUCCEAT intervention act as role models and use MI to enable the parents to be self‐compassionate and to care for themselves (Schulz von Thun, 1981; Sepulveda, Lopez, Todd, Whitaker, & Treasure, 2008). The parents learn how to apply MI: to express empathy, to motivate change through discrepancy, to roll with resistance, and to support self‐efficacy (Sepulveda, Lopez, Macdonald, & Treasure, 2008).

The SUCCEAT intervention is based upon the cognitive‐interpersonal maintenance model of AN (Schmidt & Treasure, 2006; Treasure & Schmidt, 2013). This model proposes that the following factors cause and maintain EDs: (1.) obsessive–compulsive personality traits, (2.) avoidance as a personality characteristic, (3.) positive beliefs about the illness, and (4.) responses of close others.

1. Obsessive–compulsive personality traits, such as perfectionism and rigidity in patients with AN, can lead to attention to details and a fear of making mistakes, which may cause the illness to be maintained (Crane, Roberts, & Treasure, 2007; Swinbourne et al., 2012; Treasure et al., 2008).
2. Patients with AN tend to avoid experiences, intense emotions, and close relationships (Krug et al., 2012; Russell, Schmidt, Doherty, Young, & Tchanturia, 2009; Zabala et al., 2009). As a result of the loss of brain power, starvation may increase avoidance and rigidity (Anderluh, Tchanturia, Rabe‐Hesketh, & Treasure, 2003; Cardi et al., 2017; Cardi, Di Matteo, Corfield, & Treasure, 2012; Davies, Schmidt, Stahl, & Tchanturia, 2011; Sternheim et al., 2012; Treasure & Russell, 2011; Treasure, Sepulveda, et al., 2007). Some parents share obsessive–compulsive and avoidant personality traits (Fassino et al., 2002; Treasure et al., 2008; Treasure & Cardi, 2017; Ward et al., 2001). As parents often serve as a role model for their children, SUCCEAT teaches the parents how to show flexible behaviour (Harrison, Tchanturia, Naumann, & Treasure, 2012; Roberts, Tchanturia, Stahl, Southgate, & Treasure, 2007), to pay less attention to details (Goddard, Raenker, et al., 2013), and to overcome the fear of making mistakes (Goddard, Raenker, et al., 2013; Kanakam, Raoult, Collier, & Treasure, 2012).
3. Many patients with AN have positive beliefs about their illness. The patients believe that the illness helps them to feel in control, to feel special and powerful, to cope with emotions, and to have a sense of autonomy (Branch & Eurman, 1980; Serpell, Teasdale, Troop, & Treasure, 2004). To address these positive beliefs, parents are equipped with skills to help their children to reach these positive feelings (control, autonomy, etc.) by other means than the ED (Treasure, 2016b).
4. Several distressing ED symptoms make the parents anxious (Treasure et al., 2008). Parents sometimes try to reduce their anxiety by using HEE. Animal metaphors are used to reflect dysfunctional interaction styles (Sepulveda, Lopez, Macdonald, et al., 2008). SUCCEAT emphasises the importance of collaborative caregiving. Parents are equipped with health promoting skills, function as partners of the professionals, and work like cotherapists towards patients' recovery (Macdonald, Murray, Goddard, & Treasure, 2011; Sepulveda, Lopez, Todd, et al., 2008). Carers are taught how to give just enough guidance and to care for the patient with warmth, compassion, and consistency (Gilbert, 2009).

Some ED behaviours of the patients have become automated habits (e.g., over‐exercising, vomiting, and fasting) and are no longer related to goals (Nazar et al., 2017), and some patients use the ED behaviours to deal with stressful situations (Treasure et al., 2008). One strategy to change difficult behaviours is the ABC model (Ellis, 1973). The ABC model from cognitive behavioural therapy enables the parents to help the patients change their difficult behaviours by changing antecedents (e.g., creating a comfortable atmosphere before dinner), changing their own behaviour (e.g., talking about pleasant themes during dinner), and consequences (e.g., going for a walk together after dinner). Another strategy to change difficult behaviours is changing it according to the Transtheoretical Model of Change (Prochaska & DiClemente, 1984). Carers are trained to identify which stage of change they are in themselves and which stage the patient is in (Treasure, 2016a; Whitney, Currin, Murray, & Treasure, 2012). Parents are taught how to support change in the different stages (Treasure, Sepulveda, et al., 2007). The SUCCEAT intervention informs the parents that patients sometimes relapse and need to go through repeated cycles of the stages of change (Hibbs, Magill, et al., 2015; McKnight & Boughton, 2009; Taborelli et al., 2016). The different theoretical models combine intrapersonal and interpersonal factors, as well as knowledge and skills, that can help parents to manage their own reaction to the illness and to provide an environment that is conducive to change (Treasure et al., 2010).

### 1.2 The SUCCEAT trial aims

There are three main goals of the SUCCEAT intervention: first, to increase the well‐being of the carers in order to reduce their own risk of developing a mental disorder; second, to support children's and adolescents' recovery from EDs; and third, to attain long‐term changes in the communication within the families to prevent patients from relapsing. A long‐term goal is to provide low‐threshold, cost‐effective, time‐efficient, and transregional support for carers in the future.

### 1.3 Hypotheses

The outcome measures are mentioned in brackets. These outcome measures are described in more detail in Table 2 and in Supplement S1.

**Table 2:** Outcome measures of the RCT

Instrument	Abbrev.	Domain	Purpose
General Health Questionnaire (Linden et al., 1996)	GHQ	Psychological morbidity and distress^a^	PO, C
Eating Disorder Examination (Hilbert & Tuschen‐Caffier, 2006)	EDE	ED symptomatology^b^	PO, P
Eating Disorder Inventory‐2 (Paul & Thiel, 2005)	EDI‐2	Various dimensions of EDs^a^	PO, P
Caregiver Skills (Hibbs, Rhind, Salerno, et al., 2015, c)	CASK	Skills to support a child with an ED^a^	SO, C
Experience of Caregiving Inventory (Szmukler et al., 1996, c)	ECI	Experiences in caring^a^	SO, C
Eating Disorder Symptom Impact Scale (Sepulveda, Whitney, Hankins, & Treasure, 2008, c)	EDSIS	Burden concerning caring for a child with an ED^a^	SO, C
Carers' Needs Assessment Measure (Graap et al., 2005)	CaNAM	Unmet needs respective caring^a^	SO, C
Symptom Checklist 90 (Franke, 2002)	SCL‐90	Psychiatric symptomatology^a^	SO, C
Beck Depression Inventory‐II (Hautzinger, Keller, & Kühner, 2006)	BDI‐II	Depressive symptomatology^a^	SO, C
State‐Trait‐Anxiety Inventory (Laux, Glanzmann, Schaffner, & Spielberger, 1981)	STAI	State and trait anxiety^a^	SO, C
Family Questionnaire (Wiedemann, Rayki, Feinstein, & Hahlweg, 2002)	FQ	High expressed emotions^a^	SO, C
University of Rhode Island Change Assessment Scale (Hasler, Klanghofer, & Buddeberg, 2003)	FEVER	Readiness for change^a^	SO, C
Body Mass Index (clinical measures of height and weight)	BMI	Weight status^a^	SO, P
Youth Self‐Report (Arbeitsgruppe Deutsche Child Behavior Checklist, 1998)	YSR	Behavioural, emotional problems^a^	SO, P
Health‐Related Quality of Life Questionnaire for Children and Adolescents (Ravens‐Sieberer & Bullinger, 1998a, 1998b)	KINDL	Quality of life^a^	SO, P
Family Emotional Involvement and Criticism Scale (Kronmüller et al., 2001)	FEIWK	Perceived HEE^a^	SO, P
Anorexia Nervosa Stages of Change Questionnaire (Rieger, Touyz, & Beumont, 2002, c)	ANSOCQ	Readiness to recover from AN^a^	SO, P
SUCCEAT Treatment Satisfaction Questionnaire		Feasibility and acceptability of the SUCCEAT intervention	C, Process evaluation
Client Sociodemographic and Service Receipt Inventory (Roick et al., 2001)	CSSRI	Cost‐effectiveness	C, P Economic evaluation

*Note*. SUCCEAT = Supporting Carers of Children and Adolescents with Eating Disorders in Austria; RCT = randomised controlled trial; HEE = high expressed emotions; AN = anorexia nervosa; PO = primary outcome; SO = secondary outcome; C = carers' assessment; P = patients' assessment; ED = eating disorder;

a Self‐report measure.

b Semistructured interview.

c German translation of our research group with back translations by an English native‐speaking psychologist.

#### 1.3.1 Primary hypothesis—Carers

1. Carers participating in the SUCCEAT intervention groups will report a greater reduction of distress (General Health Questionnaire: Linden et al., 1996) at postintervention (T1) than the carers in the control group.

#### 1.3.2 Primary hypothesis—Patients

- Patients whose carers participate in the SUCCEAT intervention groups will show a greater reduction of ED symptoms (Eating Disorder Examination: Hilbert & Tuschen‐Caffier, 2006; Eating Disorder Inventory 2: Paul & Thiel, 2005) at postintervention (T1) than the patients in the control group.

#### 1.3.3 Secondary hypotheses

- There will be differences in improvement of carers' and patients' outcome variables between the two SUCCEAT intervention groups (workshop support groups vs. web‐based support groups).
- Carers participating in the SUCCEAT intervention groups will report a greater reduction of burden (Eating Disorder Symptom Impact Scale: Sepulveda, Whitney, et al., 2008), as well as psychiatric symptomatology (Symptom Checklist 90: Franke, 2002), anxiety (State–Trait Anxiety Inventory: Laux et al., 1981), depression (Beck Depression Inventory II: Hautzinger et al., 2006), HEE (Family Questionnaire: Wiedemann et al., 2002), and unmet needs (Carers' Needs Assessment Measure: Graap et al., 2005) at postintervention (T1) than the carers in the control group. Further, they will report a greater increase of motivation to change (University of Rhode Island Change Assessment Scale: Hasler et al., 2003), positive experiences of caregiving (Experience of Caregiving Inventory: Szmukler et al., 1996), and caregiving skills (Caregiver Skills: Hibbs, Rhind, Salerno, et al., 2015) at postintervention (T1) than the carers in the control group.
- Carers will report acceptability and satisfaction with the SUCCEAT intervention (SUCCEAT Treatment Satisfaction Questionnaire).
- Patients whose carers participate in the SUCCEAT intervention groups will show a greater reduction of behavioural and emotional problems (Youth Self‐Report: Arbeitsgruppe Deutsche Child Behavior Checklist, 1998) and perceived HEE (Family Emotional Involvement and Criticism Scale: Kronmüller et al., 2001), as well as a greater increase of Body Mass Index, quality of life (Health‐Related Quality of Life Questionnaire for Children and Adolescents: Ravens‐Sieberer & Bullinger, 1998a, 1998b), and motivation to change (Anorexia Nervosa Stages of Change Questionnaire: Rieger et al., 2002) at postintervention (T1) than the patients in the control group.
- Outcome variables of the carers participating in the SUCCEAT intervention groups and their patients will remain stable at follow‐up (T2) compared with postintervention (T1).
- The costs of support (Client Sociodemographic and Service Receipt Inventory: Roick et al., 2001) will be lower for carers (and their patients) participating in the SUCCEAT intervention groups at postintervention (T1) and at follow‐up (T2) than for the carers (and their patients) in the control group.

## 2 METHODS

### 2.1 Trial design

**Figure 1:**
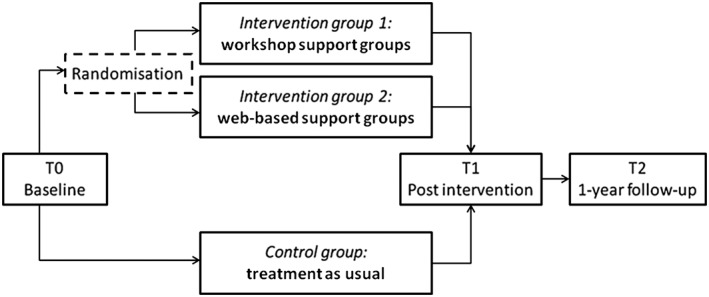
Diagram of the trial design of the intervention: Supporting Carers of Children and Adolescents with Eating Disorders in Austria

SUCCEAT is a two‐arm multicentre, parallel group RCT with a nonrandomised control group (see Figure 1). Incoming patients are screened for AN or atypical AN and are randomly allocated to the Intervention group 1 (workshop support groups) or the Intervention group 2 (web‐based support groups), respectively, in addition to treatment as usual (TAU), and compared with a nonrandomised control group (TAU). The study is conducted in Austria and Germany.

### 2.2 Participants

#### 2.2.1 Eligibility criteria for participants

The following are the inclusion criteria for children and adolescents:

- Age between 10 and 19 years
- Diagnosis of AN (F50.0) or atypical AN (F50.1) according to the *International Classification of Diseases* (World Health Organization, 1990)
- Fluent German in order to answer outcome questionnaires
- Ability to provide informed consent
- Children and adolescents have to receive TAU according to the NICE guidelines (NICE, 2017). TAU is multidisciplinary, coordinated between services, and includes psychological treatment, psychoeducation, and monitoring of weight, mental, and physical health. Psychological treatment for children and young people includes sessions without and together with their family members (for further information, see Section 2.3.2). The frequency and duration of the psychological treatment for the patients depend on their needs. Dietary counselling and medication for the patients are only offered as part of a multidisciplinary approach. For patients with an acute mental health risk, psychiatric inpatient care is considered. Patients whose physical health is severely compromised are admitted to inpatient or day‐patient service for medical stabilisation and to initiate refeeding, if these cannot be done in an outpatient setting.

The following are the exclusion criteria for children and adolescents:

- Severe comorbidity at baseline (e.g., psychosis)
- No main carer (e.g., live in a children's home with no contact to own family)

The following are the inclusion criteria for main carers who complete outcome assessment:

- Ability to provide informed consent
- The main carer has to speak German fluently in order to answer the outcome questionnaires. The main carer is the one who spends the most time with the patient. Only the main carer will answer the questionnaires about the outcome measures. Besides the main carer, other family members and carers are also invited to take part in the intervention.

The following are the exclusion criteria for main carers:

- Severe morbidity at baseline, which prevents carers from participating in the RCT (e.g., acute inpatient treatment because of physical illness)
- Carers without internet access (85% of Austrian and 92% of German households have internet access; European Commission, 2016)

#### 2.2.2 Study settings and locations where the data will be collected

Patients and carers will be recruited at different inpatient and outpatient centres with ED units, where they receive TAU. Carers and patients who meet the eligibility criteria are invited to participate in the study. Patients and carers both give their informed consent, and parents give their informed consent for children who are minors.

Carers and patients of the two SUCCEAT intervention groups will be recruited at the Medical University of Vienna, Department of Child and Adolescent Psychiatry, and by consultant child and adolescent psychiatrists and at different centres with ED units in adjacent federal states to Vienna. Patients and carers in the two intervention groups give their informed consent and complete the questionnaires at the Medical University of Vienna, Department of Child and Adolescent Psychiatry.

For the control group, patients and their carers will be recruited and receive TAU at three centres: at the Wilhelminenspital in Vienna, Department of Child and Adolescent Medicine, Inpatient and Outpatient Unit for Children and Adolescents; at the Clinical Centre Klagenfurt, Department of Neurology and Psychiatry for Children and Adolescents; and at the Parkland‐Clinic for Psychosomatic and Psychotherapy in Bad Wildungen, Germany. Patients and carers in the control group give their informed consent and complete the questionnaires at the recruiting site.

The interventions started in November 2014 and will continue approximately until April 2018. Follow‐up assessments for the remaining patients are expected to be completed by April 2019. The main ethics approval has been granted by the Medical University of Vienna Ethics Committee (1840/2013). Site‐specific ethics approvals are granted for all participating sites.

### 2.3 Treatment arms

#### 2.3.1 SUCCEAT intervention

The SUCCEAT intervention consists of eight weekly workshop sessions and web‐based modules respectively (see Table 1 for content), a SUCCEAT workbook, and the DVD *How to Care for Someone with an Eating Disorder* (The Succeed Foundation, 2011; German translation by our research group). The SUCCEAT workshop support groups and SUCCEAT web‐based support groups consist of the same content and are designed to encourage the carers to read the materials, watch the DVD, and try new behaviours as “experiments” at home. The contents of the intervention and the DVD were translated into the German language and adapted to a German‐speaking cultural context (e.g., German names of actors and meals and topics of the conversations: lyrics from a German singer) prior to the study by the SUCCEAT research group. The audio track of the DVD *How to Care for Someone with an Eating Disorder* (The Succeed Foundation, 2011) has been synchronised to the German language, and the texts have been translated. Almost all of the scenes in the DVD show young patients who still live with their parents. Therefore, the DVD is applicable to the carers of adolescent patients in our RCT. One scene shows a young adult patient (who already lives with her partner), but the content of the scene (over‐exercising) is also applicable to patients who are minors. Carers of patients with ED (who were not included in the study) tested the German materials prior to the study and gave us feedback. The DVD shows examples for communication with HEE and the same scenarios with good communication incorporating change (e.g., a family with a child with AN in a restaurant and shopping for food with a daughter suffering from AN).

Both intervention groups (workshop support groups and web‐based support groups) will involve six subgroups. Each subgroup will include the carers of about eight patients. The same two professional SUCCEAT coaches guide all the carers in each of the SUCCEAT workshop support groups and in the SUCCEAT web‐based support groups. One coach is a consultant psychiatrist, child and adolescent psychiatrist, and psychotherapist; and the other coach is a senior registrar in child and adolescent psychiatry and a psychologist. The two coaches were trained in a 2‐day workshop by Gill Todd (e.g., Salerno, Rhind, Hibbs, Micali, Schmidt, Gowers, Macdonald, Goddard, Todd, Tchanturia, et al., 2016b), who is experienced in working with carers of patients with ED applying “The New Maudsley Method” (Treasure et al., 2010; Treasure, Smith, et al., 2007).

##### SUCCEAT Intervention group 1—Workshop support groups for carers

All SUCCEAT workshop support groups are held at the Medical University of Vienna, Department of Child and Adolescent Psychiatry, once in a week in the evening (from 6 p.m. to 8 p.m.) and consist of eight weekly workshop sessions (see Table 1). Carers receive the SUCCEAT workbook as a hardcopy and the DVD (The Succeed Foundation, 2011; German translation of our research group) at the first workshop session. The two SUCCEAT coaches present the contents of the SUCCEAT intervention listed in Table 1 with a PowerPoint presentation and support the carers personally during the sessions. In each session, the carers will get a hardcopy handout with a summary of the workshop session. The workshops are designed to be interactive with role plays and group work for the carers. The carers are encouraged to talk about their concerns and experiences with each other. They have time to report their “experiments” and to reflect together.

##### SUCCEAT Intervention group 2—Web‐based support groups for carers

All the carers get to know the two SUCCEAT coaches and the other participating carers of their web‐based support group personally in a welcome meeting at the beginning of the intervention at the Medical University of Vienna, Department of Child and Adolescent Psychiatry, and subsequently complete the programme at home via Internet. The web‐based support groups consist of eight weekly web‐based modules (see Table 1). The SUCCEAT web‐based modules and the SUCCEAT workbook are available on a website within a structured program (http://www.succeat.at). The formal conditions are introduced at the welcome meeting, and the carers receive the DVD (The Succeed Foundation, 2011; German translation of our research group). Carers get access to one new web‐based module weekly. They are instructed how to work on the program at any time. Within the modules, carers fill in interactive fields of the exercises and report on their experiences and the “experiments” they carried out at home. In addition, carers are invited to use the opportunity to contact the coaches via a personal message system on the website. The coaches offer their support and give weekly feedback to the exercises and the “experiments” of the carers via the personal message system. The carers have the additional possibility to communicate with the other carers and to discuss different topics in an unguided online forum on the website. This online forum is controlled regularly by the coaches and the coaches delete disturbing comments.

#### 2.3.2 Group 3 Control group—Conventional carers' support and TAU

Carers and patients receive TAU at inpatient and outpatient centres with ED units according to the NICE guidelines (NICE, 2017). Therefore, carers take part in conventional carers' support groups or in other treatments for families (e.g., psychotherapy, medical counselling, and psychoeducation). When patients with AN are weighed at specialist services, the results are shared with their family members or carers (if appropriate). Family members or carers (as appropriate) are included in any dietary education or meal planning for the patients. Psychological treatment of the patients includes some family sessions together with their family members and psychoeducation. The therapy emphasises the role of the family in helping the person to recover. Early in treatment, a good therapeutic alliance with the parents or carers and other family members is established and the parents or carers are supported to take a central role in helping the patients manage their eating. In the second phase, parents and carers help to support the patients to establish a level of independence appropriate for their level of development. The families' concerns are addressed and family members are supported in order to cope with distress.

### 2.4 Outcome measures

To describe the sample, clinical data and demographic information about the patients and their carers are collected at baseline (T0; age, sex, educational level, family situation, migration status, and socio‐economic status). Further assessments for the primary and secondary outcome measures (see Table 2) will take place before starting the intervention (T0), after 3 months (postintervention, T1), and 1 year after the intervention (follow‐up, T2). The semistructured interview of the Eating Disorder Examination (Fairburn & Cooper, 1993; German version: Hilbert & Tuschen‐Caffier, 2006) is conducted face‐to‐face or via telephone by professionals (clinical and health psychologists and psychotherapists). These professionals also administer the other questionnaires, which are answered in a paper–pencil version by the carers and patients themselves. These professionals have already been trained to use these measures before this RCT (by experienced professionals in this field) or received training by G. W. prior to the study. The questionnaires for the process evaluation are answered by the carers of the SUCCEAT intervention after each workshop session or web‐based module and at T1 and T2. Table 2 and Supplement S1 contain further information about the questionnaires.

### 2.5 Sample size calculations

Power analysis was done with G*Power version 3.1.5. In order to detect a difference for the primary outcome (General Health Questionnaire, see Sepulveda, Lopez, Todd, et al., 2008) by calculating repeated measures analysis of variance (ANOVA; if the s*phericity* assumption is not met) at a 5% significance level with 0.88 power, carers of 40 patients per group are needed to detect medium effects (Faul, Erdfelder, Lang, & Buchner, 2007). We expect a dropout rate of about 17% (see Wagner et al., 2015). Therefore, we plan to include the carers of 48 patients per treatment arm, resulting in a total sample size of 144.

### 2.6 Randomisation

After recruitment of the patients and their carers (at the Medical University of Vienna, Department of Child and Adolescent Psychiatry, and by consultant child and adolescent psychiatrists in adjacent federal states to Vienna), patients and carers who meet the eligibility criteria give their informed consent, perform baseline assessments, and are randomly allocated to one of the two intervention groups (SUCCEAT workshop support group or SUCCEAT web‐based support group) by our research group at the Medical University of Vienna. Random allocation is made in a blockwise manner in order to keep the sizes of treatment groups similar. Incoming patients are numbered consecutively, and randomisation is done blockwise according to the consecutive numbering, in blocks of eight participants in the workshop support group and web‐based support group, respectively. The control group is not randomised.

### 2.7 Blinding

The clinical and health psychologists and psychotherapists, who conduct the assessments, are not blinded to the treatment arms.

### 2.8 Statistical analysis plan

The treatment effect will be analysed by using mixed‐design ANOVAs. The time points will be included as within group factor, and the group (workshop support group vs. web‐based support group vs. TAU) will be included as between group factor. *t* tests for dependent and independent samples will also be used in order to explore differences between specific groups and specific time points.

Two analysis strategies will be applied to account for missing data: Intention‐to‐treat analysis will be used as the primary analysis strategy. If posttreatment or follow‐up data are missing, the last‐value‐put‐forward method will be used. Additionally, the analyses will be repeated by including completers only. Completers will be defined by attending more than 50% of the workshop sessions or web‐based modules. To analyse the potential effect of treatment moderators, additional ANOVAs will be calculated including patient's age and duration of illness as covariates.

## 3 DISCUSSION

### 3.1 Limitations

On account of a research gap, some of the questionnaires that we use in this RCT are not validated in the German language yet, and there were no resources to validate them prior to this RCT. We found it important to translate and use these questionnaires because there are no comparable German versions. Although we translated these with back translations, we will validate these questionnaires in the course of this trial and calculate the psychometric properties of the questionnaires.

### 3.2 Generalisability

This study protocol describes an RCT of the SUCCEAT intervention, an intervention for carers of children and adolescents with AN and atypical AN. Several interventions show improvement for carers of adult patients with an ED (Goddard, Hibbs, et al., 2013; Grover, Naumann, et al., 2011; Hibbs, Magill, et al., 2015; Hibbs, Rhind, Leppanen, et al., 2015; Whitney, Murphy, et al., 2012). Still, little is known about the outcome of carers of children and adolescent patients (in contrast to adults) and also about the outcome of the affected children and adolescents themselves (Hibbs, Rhind, Leppanen, et al., 2015; Hoyle et al., 2013). Both of these issues are addressed in this RCT.

The SUCCEAT intervention has several further strengths in terms of carer's interventions. The feasibility and acceptability measures are included, which is important because we adapted the materials (Schmidt & Treasure, 2006; Treasure, Smith, et al., 2007; Treasure et al., 2010; Treasure & Nazar, 2016; Treasure & Schmidt, 2013) to be used with adolescents, as workshop support groups and web‐based support groups, and to be used in the German language. Above all, the SUCCEAT study will include eight families per SUCCEAT workshop support group and SUCCEAT web‐based support group in order to be even more cost‐effective than a previous study with two families per workshop (Whitney, Murphy, et al., 2012). Moreover, the content of the SUCCEAT groups is more structured than in previous workshop studies (Whitney, Murphy, et al., 2012), which facilitates replicability. Furthermore, we analyse the benefits of the DVD *How to Care for Someone with an Eating Disorder* (The Succeed Foundation, 2011) for the first time.

In addition, the SUCCEAT intervention concentrates more on MI than cognitive behavioural therapy (Grover, Naumann, et al., 2011), giving parents more time to practise communication skills, as there is evidence that therapy without a focus on weight and shape concerns can also be effective, and MI can be helpful with appealing to the patient (Pike, Walsh, Vitousek, Wilson, & Bauer, 2003; Schmidt & Treasure, 2006; Serfaty, Turkington, Heap, Ledsham, & Jolley, 1999). The content of the SUCCEAT intervention is similar to the self‐help ECHO intervention (Rhind et al., 2014), but the contents are delivered in a therapeutic form (as workshop support groups and web‐based support groups). The carers in the SUCCEAT intervention receive guidance from professional coaches, who are trained in MI and have extensive experience with EDs and other mental illnesses, in contrast to the earlier studies, where carers help other carers (ECHO; Goddard, Raenker, et al., 2013; Magill et al., 2016).

Furthermore, workshop support groups and web‐based support groups based on “The New Maudsley Method” (Treasure, Smith, et al., 2007; Treasure et al., 2010) are compared for the first time. On the one hand, the workshop support groups enable face‐to‐face contact with coaches and other carers. Some people might prefer learning in face‐to‐face settings (Williams, 2003). On the other hand, the SUCCEAT web‐based support groups are low threshold and popular. Web‐based support avoids the intimacy and stigmatization of psychotherapy, increases self‐efficacy, intensifies learning effects, and can be as effective as face‐to‐face support (Wagner et al., 2013; Williams, 2003). Our findings will show how the efficacy and acceptability of the SUCCEAT workshop support groups differ from the SUCCEAT web‐based support groups.

There is online support for patients with other mental disorders that shows positive effects: for parents of children with autism spectrum disorders (Clifford & Minnes, 2013), for parents of socially anxious youth (Reuland & Teachman, 2014), for parents to prevent anxiety problems in young children (Morgan et al., 2015), and for bipolar parents of young children (Jones et al., 2015).

Some of the themes of the SUCCEAT intervention could contribute to future interventions for carers and patients with several disorders, for example, how to deal with perfectionism, to improve emotional intelligence, and to reduce HEE. Results from this investigation will show whether the SUCCEAT intervention may be an effective, acceptable, feasible, affordable, time‐efficient, and transregional form of support for carers in the future.

## TRIAL REGISTRATION

ClinicalTrials.gov Identifier: NCT02480907.

## FUNDING

The study is funded by “Gemeinsame Gesundheitsziele aus dem Rahmen‐Pharmavertrag/Pharma Master Agreement” (a cooperation between the Austrian pharmaceutical industry and the Austrian social insurance)—Grant 99901002500 given to A. K. and G. W.

## Supporting information


**Supplement S1** Detailed description of the outcome measures used in the RCT
