# Supplementary material for: Supporting Carers of Children and Adolescents with Eating Disorders in Austria (SUCCEAT): Study protocol for a randomised controlled trial
Source: Eur Eat Disord Rev. 2018 May 6;26(5):447–61. doi: 10.1002/erv.2600 (PMC6175075; doi:10.1002/erv.2600)
Supplement: Supplementary file 1 — Supplement S1 Detailed description of the outcome measures used in the RCT [file ERV-26-447-s001.pdf]

Journal: European Eating Disorders Review DOI: 10.1002/erv.2600

Supplement S1

Detailed description of the outcome measures used in the randomised controlled trial SUCCEAT

Title:

Supporting Carers of Children and Adolescents with Eating Disorders in Austria (SUCCEAT): Study protocol for a randomised controlled trial

Authors:

Claudia Franta[1], Julia Philipp[1], Karin Waldherr[2], Stefanie Truttmann[1], Elisabeth Merl[1], Gabriele Schöfbeck[1], Doris Koubek[1], Clarissa Laczkovics[1], Hartmut Imgart[3], Annika Zanko[3], Michael Zeiler[1], Janet Treasure[4], Andreas Karwautz[1] & Gudrun Wagner[1]

Affiliations:

[1]Eating Disorders Unit, Department of Child and Adolescent Psychiatry, Medical University of Vienna, Vienna, Austria

[2]Ferdinand Porsche Distance Learning University for Applied Sciences, Vienna, Austria

[3]Parkland-Clinic, Clinic for Psychosomatic Medicine and Psychotherapy, Bad Wildungen-Reinhardshausen, Germany

[4]Department of Psychological Medicine, Section of Eating Disorders, King's College London, Institute of Psychiatry, Psychology & Neuroscience, London, UK

Address where the work was carried out and correspondence address

Ass.-Prof. Mag. Dr. Gudrun Wagner, Univ.-Prof. Dr. Andreas Karwautz, Eating Disorders Unit, Department of Child and Adolescent Psychiatry, Medical University of Vienna,
Waehringer Guertel 18-20, A-1090 Vienna, Austria, Phone: 0043-1-40400-30170;
FAX: 0043-1-9147317; E-Mail: gudrun.wagner@meduniwien.ac.at;
andreas.karwautz@meduniwien.ac.at

Primary outcome – Carers' assessment

- The General Health Questionnaire (GHQ; Goldberg, & Williams, 1988; German version: Linden et al., 1996) is an outcome measure for carers' psychological morbidity and distress. It is a well-validated 12-item self-report questionnaire using a four-point Likert scale. It is a quick, reliable (Cronbach's α = 0.91) and sensitive short form (sensitivity and specificity values of 0.68 and 0.70, respectively) with examined psychometric properties of the German version (Schmitz, Kruse & Tress, 1999).

Primary outcome – Patients' assessment

- The Eating Disorder Examination (EDE; Fairburn & Cooper, 1993; German version: Hilbert & Tuschen-Caffier, 2006) is a semi-structured interview conducted by a clinician, assessing ED symptoms over lifetime and particularly over the previous six month. The EDE is rated through the use of four subscales and a global score. Four subscales including 22 items are: 'restraint', 'eating concern', 'shape concern', and 'weight concern'. A further 14 items diagnose the ED. The German version of the EDE shows good interrater-reliability (items: $r > 0.90$, subscales: $0.92 \leq r \leq 0.99$, total score: $r = 0.99$), internal consistency (subscales: $0.73 \leq$ Cronbach's $\alpha \leq 0.86$, total score: Cronbach's $\alpha = 0.93$) and convergent validity ($0.56 \leq r \leq 0.81$).
- The Eating Disorder Inventory-2 (EDI-2; Garner, 1991; German version: Paul & Thiel, 2005) is a widely used and standardized self-report measure of symptoms related to AN. It consists of 11 subscales derived from 91 items presented in a six-point, forced choice format. Respondents answer whether each item applies 'always', 'usually', 'often', 'sometimes', 'rarely', or 'never'. Three of the subscales assess attitudes and behaviours concerning eating, weight, and shape ('drive for thinness', 'bulimia', 'body dissatisfaction'), and the remaining eight tap more general psychological traits clinically relevant to EDs ('ineffectiveness', 'perfection', 'interpersonal distrust', 'interceptive awareness', 'maturity fears', 'asceticism',

‘impulse regulation’, and ‘social insecurity’). For the German version excellent data concerning validity (Salbach-Andrae, 2010) and reliability (subscales: Cronbach’s $\alpha \geq 0.70$, total score: $0.90 \leq$ Cronbach’s $\alpha \leq 0.98$) are available (Rathner & Waldherr, 1997) with a recent standardization (Kappel et al., 2012).

Secondary outcomes – Carers’ assessments

- The questionnaire to evaluate Caregiver Skills (CASK; Hibbs et al., 2015; German translation of our research group with back translations by an English native speaking psychologist) measures the subscales: ‘bigger picture’, ‘frustration tolerance’, ‘self-care’, ‘biting-your-tongue’, ‘insight and acceptance’, and ‘emotional intelligence’. This questionnaire measures caregiver skills that may be helpful in the support of people with AN in line with the cognitive-interpersonal maintenance model (Schmidt & Treasure, 2006). Each item is scored with a visual analogue scale, with anchors 0 and 100. All standardized factor loadings of the items into their correspondent latent construct were statistically significant (at least $p < 0.05$), ranging between 0.28 and 0.84 with a mean of 0.66. All six factors have high internal consistency (subscales: $0.71 \leq$ Cronbach’s $\alpha \leq 0.85$, total score: $\alpha = 0.92$). Significant clinically relevant correlations were found between the CASK scales and other standardised measures of caregivers’ attitudes and behaviours. The CASK is sensitive to change following skills-based caregiver interventions.
- The Experience of Caregiving Inventory (ECI; Szmukler et al., 1996; German translation of our research group with back translations by an English native speaking psychologist) is a self-report measure. Eight negative subscales (‘difficult behaviours’, ‘negative symptoms’, ‘stigma’, ‘problems with services’, ‘effects on family’, ‘need to back-up’, ‘dependency’, ‘loss’) and two positive subscales (‘positive personal experiences’, ‘good aspects of relationship’) are outcome measures for interventions promoting caregivers well-being. The items explore general aspects of the experience of caring for a relative with a mental illness and use a

Likert scale from 0 to 4. Each scale has satisfactory reliability (0.74 ≤ Cronbach's α ≤ 0.91). The ECI shows good construct validity (Joyce, Leese & Szmukler, 2000).

- The Eating Disorder Symptom Impact Scale (EDSIS; Sepulveda et al. 2008; German translation of our research group with back translations by an English native speaking psychologist) measures carers' perceptions of specific caregiving burden of AN. Carers report the impact of the ED symptoms and the impact of the behaviours of their ill relative on the carers' wellbeing. The 24 items use a Likert scale from 0 to 4 and contain four factors: 'nutrition', 'guilt', 'dysregulated behaviour', and 'social isolation'. These explain 58.4% of the variance in carer distress. Reliability is good (0.84 ≤ Cronbach's α ≤ 0.90) and the 'overall scale' and the 'guilt' subscale are sensitive to change following a skills training workshop.
- The Carers' Needs Assessment Measure (CaNAM; Haigh & Treasure, 2003; German version: Graap et al., 2005) is a self-report measure to assess carers' met and unmet needs in caring for someone with an ED. The measure was designed to assess carers' perceptions about the amount of support received and the amount of information received about the illness. Items for the measure were developed with a focus group of carers of people with AN; therefore it is considered that this measure has strong construct validity. In this study we use two parts of the questionnaire ('carers' needs in the area of support from other people/organizations'; 'carers' needs coping with different fields of EDs'). The English version (Whitney et al., 2007) has good reliability (0.61 ≤ Cronbach's α ≤ 0.91) and so does the German version (Hinteregger, 2008). The scaling of the questionnaire is unclear (Hinteregger, 2008), therefore we adapted the scoring system (are there needs? yes/no; degree of met needs: rarely/sometimes/often).
- The Symptom Checklist 90 (SCL-90; Derogatis & Savitz, 1999; German version: Franke, 2002) is a self-report clinical rating scale of psychiatric symptomatology. It consists of 90 items (with a Likert scale from 0 to 4) with 83 items representing nine subscales: 'somatisation' (12 items), 'obsessive-compulsive' (10 items), 'interpersonal sensitivity' (9 items), 'depression'

(13 items), ‘anxiety’ (10 items), ‘anger-hostility’ (6 items), ‘phobic anxiety’ (7 items), ‘paranoid ideation’ (6 items), and ‘psychoticism’ (10 items). Seven additional items include i.a. disturbances in appetite and sleep. The SCL-90 also utilizes three global distress indices: ‘Global Severity Index’ (GSI), ‘Positive Symptom Distress Index’ (PSDI) and ‘Positive Symptom Total’ (PST). It may be used as a measure of patient progress. Reliability in a representative German norm sample is high (subscales: 0.75 ≤ Cronbach's α ≤ 0.87, total score: Cronbach’s α = 0.97; retest reliability subscales: r_{tt} = 0.69 - 0.92; total score r_{tt} = 0.90). Various studies prove factorial, convergent and discriminant validity (Franke, 2002).

- The Beck Depression Inventory-II (BDI-II; Beck, Steer, & Brown, 1996; German version: Hautzinger, Keller, & Kühner, 2006) relates to emotional symptoms of depression such as irritability, cognitions such as guilt, and physical symptoms such as fatigue. It has 21 multiple choice items. Each item contains four statements representing different severities of the symptoms. The carer is asked to select the statement which best describes his/her subjective well-being. The BDI-II was developed in response to the American Psychiatric Association publication of the ‘Diagnostic and Statistical Manual of Mental Disorders’, Fourth Edition (American Psychiatric Association, 2000), which changed many of the diagnostic criteria for the Major Depressive Disorder. Reliability in different German samples is high (Cronbach's α ≥ 0.89, retest reliability: r_{tt} = 0.78), a range of studies confirm validity (Hautziger et al., 2006).
- The State-Trait-Anxiety Inventory (STAI; Spielberger, 1989; German version: Laux, Glanzmann, Schaffner, & Spielberger, 1981) is a commonly used measure of state (STAI G Form X1, 20 items) and trait (STAI G Form X2, 20 items) anxiety. The STAI is an indicator of caregiver distress. Items utilize a four-point Likert scale. It is a sensitive predictor of caregiver distress over time and varies with changes in support systems. Reliability is high for both scales (Cronbach's α = 0.90) and several studies confirm validity (Laux et al., 1981).
- The Family Questionnaire (FQ; German version: Wiedemann et al., 2002) is a 20-item self-report questionnaire designed to detect the emotional climate within the family (i.e. HEE).

Scores are given on a four-point Likert scale and form two subscales of HEE in carers: ‘Emotional over-involvement’ (EOI) and ‘Criticism’ (CC). Retest reliability (EOI: r_{tt} = 0.91, CC: r_{tt} = 0.84) and internal consistency (EOI: Cronbach's α = 0.82, CC: Cronbach's α = 0.92) are high. Validation studies are also successful (Wiedemann et al., 2002).

- The University of Rhode Island Change Assessment Scale (URICA; McConnaughy, Prochaska, & Velicer, 1983; German version: FEVER; Hasler, Klanghofer, & Buddeberg, 2003) measures the stages of change, based on the transtheoretical model of change (Prochaska & DiClemente, 1984). Items rate on a five-point Likert scale. People change their behaviour gradually across different stages. Scales of this questionnaire represent the carers’ readiness for change (‘precontemplation’, ‘contemplation’, and ‘action’). The German version shows good internal consistency of the scales (precontemplation: Cronbach's α = 0.72, contemplation: Cronbach's α = 0.78, action: Cronbach's α = 0.86) and there is evidence for predictive and concordant validity.

Secondary outcomes – Patients’ assessments

- The Body Mass Index (BMI) is obtained from clinical measures of height and weight.
- The Youth Self Report (YSR; Achenbach, 1991; German version: Arbeitsgruppe deutsche Child Behaviour Checklist, 1998) is a self-report measure for adolescents to rate regarding various behavioural and emotional problems. It is one of the most widely-used standardized measures in child and adolescent psychology. First, adolescents name their competencies, which build the three scales: ‘activities’, ‘social components’ and ‘school’. Then, they describe their illnesses, problems, worries and what they like about themselves in an open answer format. The next 112 items address traits and behavioural issues in adolescents, and are scored using a three-point Likert scale. The YSR yields scores on the syndrome scales: ‘anxious/depressed’, ‘withdrawn/depressed’, ‘somatic complaints’, ‘social problems’, ‘thought problems’, ‘attention problems’, ‘rule-breaking behaviour’, ‘aggressive behaviour’,

and ‘other problems’. Reliability of the German version is high (r ≥ 0.70) and many studies convey good validity (Döpfner, Berner & Lehmkuhl, 1994).

- The Health-Related Quality of Life Questionnaire for Children and Adolescents (KINDL; German version: Ravens-Sieberer & Bullinger, 1998a, 1998b) comprises of the subscales: ‘physical well-being’, ‘psychological well-being’, ‘self-worth’, ‘family’, ‘friends’, and ‘school’. It is a self-report measure. The five-point answer format for the 24 items require respondents to answer whether each item applies ‘always’, ‘often’, ‘sometimes’, ‘rarely’, or ‘never’. The German version of the questionnaire is validated and shows good psychometric properties for the scale structure (scalefit 80% for all subscales), reliability (subscales: Cronbach’s α ≈ 0.70, total score: Cronbach’s α = 0.84), factorial validity, convergent validity, discriminate validity, and sensitivity (Ravens-Sieberer & Bullinger, 1998a, 1998b).
- The Family Emotional Involvement and Criticism Scale (FEICS; Shields, Franks, Harp, Campbell, & McDaniel, 1994; German version: FEIWK; Kronmüller et al., 2001) is a questionnaire which assesses perceived HEE. ‘Emotional involvement’ and ‘criticism’ are scored over 14 items using a five-point Likert scale. The two-factor structure from the original American version was replicated in German with a good reliability (‘Emotional Involvement’: Cronbach’s α = 0.64 and ‘Criticism’: Cronbach’s α = 0.79). Good correlation between the measured factors, and clinical and other diagnostic markers particular to family pathology was found.
- The Anorexia Nervosa Stages of Change Questionnaire (ANSOCQ; Rieger, Touyz, & Beumont, 2002; German translation by our research group with back translations by a native speaking psychologist) assesses patients’ readiness to recover from AN. The questionnaire contains 20 items related to ED symptomatology (‘body satisfaction’, ‘eating behaviours’, ‘weight control’, ‘emotional problems’, ‘personality problems’, and ‘interpersonal problems’). Each item contains five statements representing the stages of change (precontemplation, contemplation, preparation, action, maintenance) and the individual is asked to select the

statement which best describes his/her attitude or behaviour regarding changing the symptom. The ANSOCQ demonstrated good internal consistency (Cronbach's α = 0.90), one week test-retest reliability (0.89) and good validity (Rieger et al., 2000).

Process evaluation

- The SUCCEAT Treatment Satisfaction Questionnaire (developed by our research group) evaluates feasibility and acceptability of the SUCCEAT intervention after each workshop session and each web-based module and also after the whole program. After each of the 8 workshop sessions or web-based modules, all participating carers (main carers and additional carers) rate understandability, comprehensiveness and utility of the session or module in our SUCCEAT Treatment Satisfaction Questionnaire on a five-point Likert scale. Two open questions additionally address favourite contents and desired improvements.

  At the end of the intervention (T1), the main carers of both intervention groups will answer questions in our SUCCEAT Treatment Satisfaction Questionnaire about following themes on a five-point Likert scale: satisfaction with the content of the workshop sessions or web-based modules in general, the coaches, the contact to other carers, usage of the SUCCEAT workbook, the DVD and an open question for further remarks.

  One year after the intervention (T2), the main carers of both intervention groups will answer questions about the following topics on a five-point Likert scale: how many other 'experiments' they tried after the intervention ended, how much additional information they read from the SUCCEAT workbook after the intervention, how often they used the DVD after the intervention, how much contact they had to other carers after the intervention, their satisfaction with SUCCEAT overall and an open question for further remarks.

Economic evaluation

- The Client Sociodemographic and Service Receipt Inventory (CSSRI; Chisholm et al., 2000; German version: Roick et al., 2001) measures cost-effectiveness. A brief version of the CSSRI was developed for this study to document main carers' and patients' use of health services. At T0 carers report their own past treatments because of the patients' ED (e.g. psychotherapist, self-help) and the patients' past inpatient and outpatient treatments because of their ED (e.g. general practitioner, paediatrician, psychiatrist, psychotherapist) and the medication of the patients over the last month because of their ED. Additionally, carers list their previous inpatient and outpatient treatments because of their own mental illnesses (e.g. inpatient treatment, psychologist). At T1 carers report the same treatments since T0 and the patients' medication over the last month because of their ED and at T2 the same treatments since T1 and the patients' medication over the last month because of their ED.

References:

Achenbach, T. M. (1991). *Manual for the Youth Self-Report and 1991 profile*. Burlington, VT: Department of Psychiatry, University of Vermont.

American Psychiatric Association (2000). *Diagnostic and statistical manual of mental disorders* (4th ed.). Washington, DC: Author.

Arbeitsgruppe Deutsche Child Behavior Checklist. (1998). *Fragebogen für Jugendliche; deutsche Bearbeitung der Youth Self-Report Form der Child Behavior Checklist (YSR). Einführung und Anleitung zur Handauswertung mit deutschen Normen, bearbeitet von M. Döpfner, J. Plück, S. Bölte, K. Lenz, P. Melchers & K. Heim (2. Aufl.).* Köln: Arbeitsgruppe Kinder-, Jugend- und Familiendiagnostik (KJFD).

Beck, A. T., Steer, R. A., Brown, G. K. (1996). *Manual for the Beck Depression Inventory–II.* San Antonio, TX: Psychological Corporation.

Chisholm, D., Knapp, M. R., Knudsen, H. C., Amaddeo, F., Gaite, L., van Wijngaarden, B.& the EPSILON Study Group. (2000). *Client Sociodemographic and Service Receipt Interview-European Version*. Br J Psychiatry, 17(39), 28-33.

Derogatis, L. R. & Savitz, K. L. (1999). The SCL-90-R, Brief Symptom Inventory, and matching clinical rating scales. In M. E. Maruish (Ed.), *The use of psychological testing for treatment planning and outcome assessment* (2nd ed.) (pp. 679-724). Mahwah, NJ: Erlbaum.

Döpfner, M., Berner, W., & Lehmkuhl, B. (1994). *Handbuch: Fragebogen für Jugendliche Forschungsergebnisse zur deutschen Fassung des Youth Self-Reports (YSR) und der Child Behavior Checklist.* Köln: Arbeitsgruppe Kinder-, Jugend- und Familiendiagnostik.

Fairburn, C. G., & Cooper, Z. (1993). The Eating Disorder Examination (12th ed.). In C. G. Fairburn & G. T. Wilson (Eds.), *Binge eating: Nature, assessment and treatment* (pp. 317-360). New York: Guilford.

Franke, G. H. (2002). *SCL-90-R - Die Symptom-Checkliste von L. R. Derogatis (2. vollständig überarbeitete und neu normierte Auflage).* Göttingen: Beltz Test.

Garner, D. M. (1991). *Eating Disorder Inventory-2. Professional Manual*. Odessa, FL: Psychological Assessment Resources (PAR).

Goldberg, D. & Williams, P. (1988). *A users guide to the General Health Questionnaire*. Slough: NFER-Nelson.

Graap, H., Bleich, S., Wilhelm, J., Herbst, F., Trostmann, Y., Wancata, J., & de Zwaan, M. (2005). Die Belastung und Bedürfnisse Angehöriger anorektischer und bulimischer PatientInnen. *Neuropsychiatrie, 19*(4), 155-161.

Haigh, R., & Treasure, J. (2003). Investigating the Needs of Carers in the Area of Eating Disorders: Development of the Carers' Needs Assessment Measure (CaNAM). *European Eating Disorders Review, 11*, 125-141. DOI: 10.1002/erv.487

Hasler, G., Klanghofer, R., & Buddeberg, C. (2003). Der Fragebogen zur Erfassung der Veränderungsbereitschaft (FEVER). Testung der deutschen Version der University of Rhode

Island Change Assessment Scale (URICA). *Psychotherapie, Psychosomatik, Medizinische Psychologie, 53*, 406-411. DOI: 10.1055/s-2003-42172

Hautzinger, M., Keller, F., & Kühner, C. (2006). *Das Beck Depressionsinventar II. Deutsche Bearbeitung und Handbuch zum BDI II.* Frankfurt a. M.: Harcourt Test Services.

Hibbs, R., Rhind, C., Salerno, L., Lo Coco, G., Goddard, E., Schmidt, U., Micali, N., Gowers, S., Beecham, J., Macdonald, P., Todd, G., Campbell, I., & Treasure, J. (2015). Development and validation of a scale to measure caregiver skills in eating disorders. *International Journal of Eating Disorders, 48*(3), 290-297. DOI: 10.1002/eat.22362

Hilbert, A., & Tuschen-Caffier, B. (2006). *Eating Disorder Examination: Deutschsprachige Übersetzung*. Münster: Verlag für Psychotherapie.

Hinteregger, M. (2008). *Psychometrische Überprüfung der deutschen Fassung des „Carers' Needs Assessment Measure (CaNAM)"* (Master´s Thesis). Retrieved February 20, 2017, from http://othes.univie.ac.at/2551/1/2008-11-03_8703893.pdf

Joyce, J., Leese, M., & Szmukler, G. (2000). The Experience of Caregiving Inventory: further evidence. *Social Psychiatry and Psychiatric Epidemiology, 35*(4), 185-189. DOI: 10.1007/s001270050202

Kappel, V., Thiel, A., Holzhausen, M., Jaite, C., Schneider, N., Pfeiffer, E., Lehmkuhl, U., & Salbach-Andrae, H. (2012). Eating Disorder Inventory-2 (EDI-2) Normierung an einer Stichprobe normalgewichtiger Schüler im Alter von 10 bis 20 Jahren und an Patientinnen mit Anorexia nervosa. *Diagnostica*, *58*(3), 127-144. DOI: 10.1026/0012-1924/a000069

Kronmüller, K. T., Krummheuer, C., Topp, F., Zipfel, S., Herzog, W., & Hartmann, M. (2001). Der Fragebogen zur familiären emotionalen Involviertheit und wahrgenommen Kritik (FEIWK). Ein Verfahren zur Erfassung von Expressed Emotion. *Psychotherapie, Psychosomatik, Medizinische Psychologie, 51*, 377-383. DOI: 10.1055/s-2001-16897

Laux, L., Glanzmann, P., Schaffner, P., & Spielberger, C. D. (1981) *Das State-Trait-Angstinventar (Testmappe mit Handanweisung, Fragebogen STAI-G Form X 1 und Fragebogen STAI-G Form X 2)*. Weinheim: Beltz

Linden, M., Maier, W., Achenberger, M., Herr, R., Helmchen, H., & Benkert, O. (1996). Psychische Erkrankungen und Ihre Behandlungen in Allgemeinarztpraxen in Deutschland. *Nervenarzt, 67*, 205-215.

McConnaughy, E. A., Prochaska, J. O. & Velicer, W. (1983). Stages of change in psychotherapy: Measurement and sample profiles. *Psychotherapy: Theory, Research, and Practice, 20*(3), 368-375. DOI: 10.1037/h0090198

Paul, T., & Thiel, A. (2005). *EDI-2. Eating Disorder Inventory-2*. Göttingen: Hogrefe.

Prochaska, J., & DiClemente, C. (1984). The Transtheoretical Approach: Crossing the Traditional Boundaries of Therapy. Homewood, IL: Dow Jones Irwin.

Rathner, G., & Waldherr, K. (1997). Eating Disorder Inventory-2: Eine deutschsprachige Validierung mit Normen für weibliche und männliche Jugendliche. *Zeitschrift für Klinische Psychologie, Psychiatrie und Psychotherapie, 45*, 157-182.

Ravens-Sieberer, U., & Bullinger, M. (1998a). Assessing health related quality of life in chronically ill children with the German KINDL: first psychometric and content-analytical results. *Quality of Life Research*, *7*, 399-407. DOI: 10.1023/A:1008853819715

Ravens-Sieberer, U., & Bullinger, M. (1998b). News from the KINDL-Questionnaire – A new version for adolescents. *Quality of Life Research, 7*, 653. DOI: 10.1023/B:QURE.0000004424.99048.86

Rieger, E., Touyz, S. W., & Beumont, P. J. V. (2002). The Anorexia Nervosa Stages of Change Questionnaire (ANSOCQ): Information regarding its psychometric properties. *International Journal of Eating Disorders, 32*(1), 24-38. DOI: 10.1002/eat.10056

Rieger, E., Touyz, S., Schotte, D., Beumont, P., Russell, L., Clarke, S., Kohn, M., & Griffiths, R. (2000). Development of an instrument to assess readiness to recover in anorexia nervosa. *International Journal of Eating Disorders, 28*, 387-396. DOI: 10.1002/1098-108X(200012)28:4<387::AID-EAT6>3.0.CO;2-Y

Roick, C., Kilian, R., Matschinger, H., Bernert, S., Mory, C., & Angermeyer, M. C. (2001). Die deutsche Version des Client Sociodemographic and Service Receipt Inventory. Ein Instrument zur Erfassung psychiatrischer Versorgungskosten. *Psychiatrische Praxis, 28*(2), 84–90.

Salbach-Andrae, H., Schneider, N., Bürger, A., Pfeiffer, E., Lehmkuhl, U., & Holzhausen, M. (2010). Psychometrische Gütekriterien des Eating Disorder Inventory (EDI-2) bei Jugendlichen. *Zeitschrift für Kinder- und Jugendpsychiatrie und Psychotherapie, 38*, 219–228. DOI: 10.1024/1422-4917/a000035

Schmitz, N., Kruse, J., & Tress, W. (1999). Psychometric properties of the General Health Questionnaire (GHQ-12) in a German primary care sample. *Acta Psychiatrica Scandinavica, 100*, 462-468.

Sepulveda, A. R., Whitney, J., Hankins, M., & Treasure, J. (2008). Development and validation of an Eating Disorders Symptom Impact Scale (EDSIS) for carers of people with eating disorders. *Health and Quality of Life Outcomes, 6*, 28-36. DOI: 10.1186/1477-7525-6-28

Shields, C. G., Franks, P., Harp, J. J., Campbell, T. L., & McDaniel, S. H. (1994). Family emotional involvement and criticism scale (FEICS): II. Reliability and validity studies. *Family Systems Medicine*, *12*(4), 361-377. DOI: 10.1037/h0089289

Spielberger, C. D. (1989). *State-Trait Anxiety Inventory: A comprehensive bibliography*. Palo Alto, CA: Consulting Psychologists Press.

Szmukler, G. I., Burgess, P., Herrman, H., Benson, A., Colusa, S., & Bloch, S. (1996). Caregiving for relatives with serious mental illness: the development of the Experience of Caregiving Inventory. *Social Psychiatry and Psychiatric Epidemiology, 31*, 137-148.

Whitney, J., Haigh, R., Weinman, J., & Treasure, J. (2007). Caring for people with eating disorders: Factors associated with psychological distress and negative caregiving appraisal in carers of

people with eating disorders. *British Journal of Clinical Psychology, 46*, 413-428. DOI: 10.1348/014466507X173781

Wiedemann, G., Rayki, O., Feinstein, E., & Hahlweg, K. (2002). The Family Questionnaire: Development and validation of a new self-report scale for assessing expressed emotion. *Psychiatry Research, 109*, 265-279. DOI: 10.1016/S0165-1781(02)00023-9
